# The smallest *Zosterophyllum* plant from the Lower Devonian of South China and the divergent life-history strategies in zosterophyllopsids

**DOI:** 10.1098/rspb.2024.2337

**Published:** 2025-01-15

**Authors:** Pu Huang, Jia-Shu Wang, Yi-Ling Wang, Lu Liu, Jing-Yu Zhao, Jin-Zhuang Xue

**Affiliations:** ^1^ State Key Laboratory of Palaeobiology and Stratigraphy, Nanjing Institute of Geology and Palaeontology, Chinese Academy of Sciences, Nanjing 210008, People’s Republic of China; ^2^ Geological Museum of China, Beijing 100034, People’s Republic of China; ^3^ The Key Laboratory of Orogenic Belts and Crustal Evolution, School of Earth and Space Sciences, Peking University, Beijing 100871, People’s Republic of China; ^4^ National Natural History Museum of China, Beijing 100050, People’s Republic of China; ^5^ School of Resources and Civil Engineering, Suzhou University, Suzhou 234000, People’s Republic of China

**Keywords:** early land plants, *Zosterophyllum baoyangense *sp. nov., Early Devonian, life-history strategies

## Abstract

Plants have evolved different life-history strategies to overcome limited amounts of available resources; however, when and how divergent strategies of sexual reproduction evolved in early land plants are not well understood. As one of the notable and vital components of early terrestrial vegetation, the Zosterophyllopsida and its type genus *Zosterophyllum* reached maximum species diversity during the Pragian (Early Devonian; *ca* 410.8–407.6 million years ago). Here we describe a new species, *Zosterophyllum baoyangense* sp. nov., based on well-preserved specimens from the Pragian-aged Mangshan Group of Duyun, Guizhou Province, China. The new plant is characterized by its small size, K-shaped branching and tiny spikes with 5–10 sporangia. This plant is most likely *r*-selected, completing its whole lifespan in a short time, and such a strategy contributes to reproduction in a suitable window time. In contrast, most other species of *Zosterophyllum* and the zosterophyllopsids on a broader scale are larger in body size and have greater investments in fertile tissues, reflected in the size and total number of sporangia. We argue that the zosterophyllopsids probably benefited from the divergence of various life-history strategies and thus constituted a major part of the Early Devonian floras.

## 1. Introduction

The evolution of life histories is strongly influenced by their ecological contexts [1]. Thus, plants have evolved different strategies to contend with limited resources [2]. Both vegetative growth and sexual reproduction occur in early land plants [3–5]. It has been demonstrated that some early plants could produce large colonies through vegetative, clonal growth [4–6], while at the same time dispersing over distance by spores, initiating the sexual phase of the life cycle [3,7]. The trade-offs between spore size and quantity in heterosporous plant reproduction are well known [7–10], but how life-history strategies evolved in homosporous early land plants is not as well understood.

The initial radiation of vascular land plants during the late Silurian to the Devonian has been regarded as the terrestrial equivalent of the Cambrian explosion of marine animals [11–14]. Novel structures such as leaves, roots, megaspores and secondary xylem evolved during this time interval, especially in the Early Devonian [10,15,16]. As a predominant component of Early Devonian floras, the plants in the genus *Zosterophyllum* (Zosterophyllopsida) are characterized by leafless axes, K- or H-shaped branching and lateral sporangia in spikes (see electronic supplementary material, tables S1 and S2; [13,16–19]). At least 20 species of *Zosterophyllum* have been reported around the world [13,20], especially from China, North America, Europe and Australia, and some species are preserved as complete or nearly complete plants, such as *Zosterophyllum shengfengense* [16,21,22]. The South China Block is one of the most important sources of early land plants and yields at least 14 species of *Zosterophyllum*. Herein, we report on a new species of *Zosterophyllum* from Guizhou Province, southwestern China, and we investigate morphological variation in the genus and its related taxa, shedding light on how divergent reproductive strategies evolved in this group.

## 2. Material and methods

### (a) Specimens

Two compression specimens were collected from the Mangshan Group at Baoyang section near Baoyang Village, Duyun City, Guizhou Province (GPS: 26°6′44″N, 107°26′44″E; see electronic supplementary material, figure S1). Specimen PB203562 is preserved in a grey to yellow mudstone of bed 6, while the other specimen, PB203563 is in a grey siltstone of bed 16. The specimens were prepared by fine needle to expose them from the matrix under light microscopy, and they were photographed using a digital camera system. The measurements were made using ImageJ 1.51 k (http://imagej.nih.gov/ij). All specimens are housed at Nanjing Institution of Geology and Palaeontology, Chinese Academy of Sciences.

The Mangshan Group at the Baoyang section mainly consists of thickly bedded quartz sandstone, with intercalated silty mudstone and argillaceous siltstone [23]. This group is unconformably underlain by the Silurian Wengxiang Formation and is overlain by the Middle Devonian Dushan Formation [23]. The plant-bearing beds within the lower part of this group were inferred to be Pragian (Early Devonian) in age, based on recently discovered macrofossil plants such as *Adoketophyton subverticillatum*, *Zosterophyllum australianum* and *Hedeia* (*Yarravia*), which are common elements in the well-known Posongchong Formation of Yunnan Province [23–25]. The latter formation was considered to be Pragian based on spore assemblages, plants and stratigraphic correlation [13].

### (b) Morphological measurements of *Zosterophyllum* species

The morphological descriptors of *Zosterophyllum* species are shown in electronic supplementary material, figure S2, including width and length of spikes, width and length of axes, sporangial height and width, etc. These descriptors are commonly used in the documentation of fossil plants and can be easily obtained.

Compared with extant plants, the fertile investment involving the total number of spores and survival rate of spores cannot be obtained from plant fossils. Here, we define a new descriptor, total sporangial accommodation (TSA), to evaluate the mass or energy investment for spore production in each plant. The TSA is estimated by the following formula for each fertile axis:


TSA=n⋅(h⋅w⋅t)

where the total number of the visible sporangia in a spike or fertile region is symbolized as *n*, and the sporangial height, width and thickness are symbolized as *h*, *w* and *t*, respectively.

The sporangial thickness is rarely observed, but it is seemingly stable as observed in a few records (such as [16,26]). Here we assume a constant value for the thickness of sporangia (0.6 mm, as measured in the well-preserved specimens of *Z. shengfengense* [16]; that is, it is assumed that *t* = 0.6 mm for all plants). For example, the spike of *Zosterophyllum qujingense* consists of 3−8 sporangia, which are 2.5−4.5 mm high and 3.1−4.7 mm wide, and therefore the value of TSA for this species is 14.0−101.5 mm^3^. When the number of visible sporangia or estimated sporangia is not documented in species descriptions, we obtained an estimation from the published illustrations in the original literature.

In addition to our specimens, data on other *Zosterophyllum* species were collected from descriptions in the original literature or were directly measured by the authors based on the published illustrations. The data sources include those of Cascales-Miñana & Meyer-Berthaud [20] and more recent works, such as in [22,27,28], spanning from Silurian to Early Devonian. However, only species of *Zosterophyllum* that have been well-studied or well-illustrated are included, while species with open nomenclature such as those with 'sp.' and 'aff.' are excluded, except for the earliest record *Zosterophyllum* sp. from the Silurian of Canada [29]. The exact assignment of species with such open nomenclature requires further work. The same species from different localities are regarded as different data points.

Plant height cannot be measured directly for most species of *Zosterophyllum*, but we could assess their possible height, or corss-compare specimens with similar levels of preservation condition. An exceptional specimen of *Z. shengfengense* provides a good illustration that the plants of this group can be classified into four parts: the subterraneous fibrous root-like axes, the semi-below or below-ground rhizome, the basal aerial axes with K- or H-shaped branching and the main erect part containing fertile regions or spikes [13,16]. Hence, the combination of the lengths of the latter two parts is regarded as representing the majority of the plant height.

Here, the plant remains of *Zosterophyllum* were divided into three groups, based on their preservation status. Most specimens are only preserved in the middle to upper fertile parts (Group I); some specimens exhibit fertile axes with K- or H-shaped branching (Group II), such as *Z. qujingense*, *Zosterophyllum minorstachyum* and our plant; and a few other specimens show complete plants, such as *Z. shengfengense*, here considered as Group III. If rhizomes and aerial fertile parts of a species are found, then although they may not be directly connected, such species are also included in Group III; this is the case for *Zosterophyllum xishanense*, *Zosterophyllum sinense* and *Zosterophyllum myretonianum*.

By following the above methods, we compiled a new dataset of morphological measurements of *Zosterophyllum* species (see electronic supplementary material, table S3). The genus *Zosterophyllum* is probably not monophyletic based on phylogenetic analyses [11,13,30]. However, it is noted here that cladistic analyses of early land plants usually suffer from limited character sampling; for example, most *Zosterophyllum* species lack features of anatomy and spores. At present, whether or not the genus *Zosterophyllum* is monophyletic remains an unresolved issue. Nevertheless, we extended the scope of our analysis to consider other zosterophyllopsids in order to provide (i) an extrapolation of our results to a higher-level category and (ii) a discussion of character evolution within a (proposed) monophyletic group.

### (c) Measurements of zosterophyllopsids (and early lycopsids)

Two different phylogenetic frameworks were employed herein (see electronic supplementary material, figure S5); the first recognized an inclusive clade, the Lycophytina *sensu* Kenrick & Crane (L-KC for short), which includes the Zosterophyllopsida *sensu* Kenrick & Crane, lycopsids and some other taxa [11], and the second recognized the Zosterophyllopsida *sensu* Hao & Xue (Z-HX for short) as a monophyletic clade [13]. By using the morphological descriptors shown in electronic supplementary material, figure S2, we obtained measurements of the members of L-KC and Z-HX. In fact, numerous zosterophyllopsids were not sampled in phylogenetic analyses [11,13,30,31], including some taxa that were recently described such as *Baoyinia* [32]. For those that have not been included in phylogenetic analyses, their assignment to the L-KC and the Z-HX was based on the authors’ own judgement. The dataset is shown in electronic supplementary material, table S4.

The species with open nomenclature were excluded, except for those from the Ludlow. The sporangiotaxis and sporangium orientation of other zosterophyllopsids are more divergent than in *Zosterophyllum*. For TSA, three types of taxa were not considered: (i) some taxa show sparsely attached sporangia, such as *Danziella artesiana*, *Forania*, *Sawdonia*, etc.; (ii) the distribution of sporangia in some taxa is scattered on the axes, and thus it is hard to discern a spike or fertile region, such as *Deheubarthia*, *Gosslingia*, etc.; (iii) the fertile regions of some taxa are not complete, such as *Crenaticaulis*, *Ventarura* etc. However, other morphological parameters of all these taxa, such as width of axes and sporangial size, were included in the analyses.

### (d) Data analyses

We assigned the sampled taxa to five time bins, based on the International Chronostratigraphic Chart v. 2023/09 (https://stratigraphy.org/chart#latest-version), that is, the Ludlow, Pridoli, Lochkovian, Pragian and Emsian, in an ascending order. The majority of *Zosterophyllum* species are usually confined within a single time bin (singletons). Species that extend across two or more time bins are rare, and our treatments are as follow. We treated the *Zosterophyllum* species from the Xujiachong Formation as Pragian age because that most plants occur in the middle to upper part of the Xujiachong Formation, although the upper part of this formation is regarded as early Pragian to earliest Emsian in age based on spore assemblages [33]. *Zosterophyllum rhenanum* and *Zosterophyllum confertum* were suggested to be derived from Lochkovian to Emsian or Pragian to Emsian deposits, but the occurrences of both species are mainly distributed in the Pragian [34,35], and thus a Pragian age was assigned to them in our dataset. For the species in the L-KC and the Z-HX, we followed the age assignments in the original literature and used a range-through method for their statistics; that is, if one species was recorded from the Lochkovian to Emsian then it was counted once separately in each of the Lochkovian, Pragian and Emsian.

Our dataset was analysed and visualized using R v.4.3.2 (RStudio/2023.09.1+494) with packages ggplot2 v.3.4.2 and ggsci v. 3.0.0 [36].

## 3. Results

### (a) Systematic palaeontology

**Class:** Zosterophyllopsida Hao & Xue [13]
**Order:** Zosterophyllales Hao & Xue [13]
**Family:** Zosterophyllaceae Banks [37]
**Genus:** *Zosterophyllum* Penhallow [17]

*Type species Z. myretonianum* Penhallow [17]

*Zosterophyllum baoyangense* Huang & Xue sp. nov.

*Etymology*. The specific epithet is derived from Baoyang Village, where the fossils were collected.

**Figure 1.**
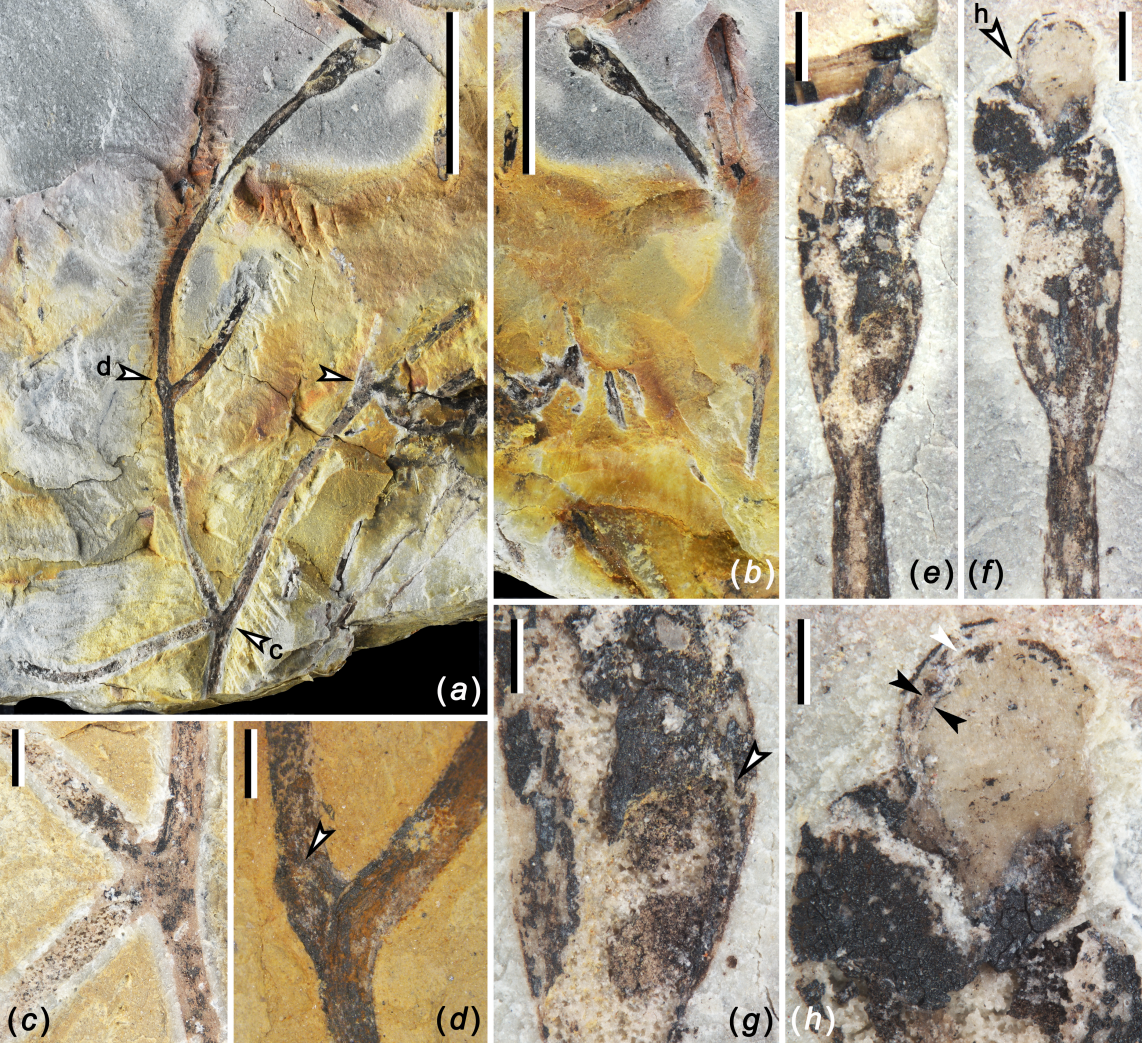
*Zosterophyllum baoyangense* sp. nov. (*a,b*) PB203562, part and counterpart, showing a fertile axis with K-shaped branching and a terminal spike. Arrows highlight branching points. The parts indicated by arrows c and d are enlarged in (*c,d*), respectively; (*c*) K-shaped branching; (*d*) branching point showing a nearby protuberance (arrow); (*e,f*) Enlarged view of the terminal spike in (*a*) and (*b*); (*g*) enlarged view of the basal part of the spike in (*e*). Arrow points to the margin of the basal sporangium. (*h*) Enlargement of the distal sporangia in (*f*) (arrow h), showing dehiscence line (white arrow) and peripheral rim along the convex distal margin (the area between two black arrows). Scale bars: (*a,b*), 10 mm; (*c–f*), 1 mm; (*g,h*), 0.5 mm.

*Holotype designated herein*. PB203562 (figure 1).

*Paratype*. PB203563 (see electronic supplementary material, figure S3).

*Locality and horizon*. Baoyang Section, Baoyang Village, Duyun City, Guizhou Province; the lower part of the Mangshan Group; Early Devonian (Pragian; see electronic supplementary material, figure S1).

*Repository*. All specimens are deposited at the Nanjing Institute of Geology and Palaeontology, Chinese Academy of Sciences, China.

*Specific diagnoses*. Rhizome with K-shaped branching. Erect axis with tiny spikes. Axes 0.5−1.3 mm wide. Spikes, 5.8−10.8 mm high and 2.0−2.8 mm in maximum width, consisting of 5–10 sporangia that are spirally arranged. Sporangia oval to semicircular, 1.6−2.0 mm high and 0.9−1.4 mm wide, departing from axis at an acute angle by a short stalk. Thin peripheral rim *ca* 80 μm wide, extending along the convex distal margin and lacking thickened dehiscence mechanism.

#### (b) Description

#### Specimen PB203562

This specimen shows smooth axes with K-shaped branching and a terminal tiny spike (figure 1). We interpret the part with K-shaped branching as the rhizome of the plant, producing an erect axis that is isotomously divided and bears a terminal spike. The axes are consistent in diameter, *ca* 0.7−0.8 mm, but swell at branching points (figure 1*a*,*c*,*d*). The rhizome first divides to form a K-shaped branch at one node (figure 1*a*, arrow c; figure 1*c*) and one of the daughter axes is 14.3 mm long and divides dichotomously, producing two axes (figure 1*a*, arrow d; figure 1*d*), one of which is terminated by a spike (figure 1*a*,*b*,*e*,*f*). The other daughter axis is 14.4 mm long and divides once, but the ends are broken (figure 1*a*, upper right arrow). A protuberance occurs above the branching point and this protuberance, *ca* 0.5 mm wide by 0.9 mm long, is possibly a subaxillary branch or a broken lateral branch (figure 1*d*). From the last branching point to the base of the spike, the fertile axis extends up to 20.5 mm in length (figure 1*a*) and the proposed erect plant body is *ca* 45.4 mm in whole length.

The spikes are short and tiny, only 5.8 mm long by 2.0 mm in maximum width (figure 1*e*,*f*). Five visible sporangia are tightly arranged in a spiral arrangement (figure 1*e*,*f*; see electronic supplementary material, figure S4*a*) and depart from the axis with an acute angle. The junction between a sporangium and its stalk is absent or not clear. The topmost sporangium in the spike is oval in face view. These sporangia measure 1.7−1.8 mm high and 0.9−1.1 mm wide (figure 1*e*,*f*,*h*). The dehiscence line is located on the distal margin, and it divides the sporangium into two valves (figure 1*h*). The peripheral rim extends along the convex distal margin (figure 1*h*, black arrows), *ca* 80 μm wide, without thickened mechanism. Anatomy of the axis and spores is unknown.

#### Specimen PB203563

The fertile axis is *ca* 25.7 mm long, and the spike is 10.8 mm long and 2.8 mm in maximum width (see electronic supplementary material, figure S3). The axis is *ca* 1.0 mm at its base and decreases to 0.5 mm wide, and then increases to 1.3 mm wide at the position just below the spike. The spike consists of ten sporangia that are spirally and compactly arranged, and the sporangia are inserted at an acute angle (see electronic supplementary material, figures S3*a–c*; S4*b*). Most sporangia are semicircular in outline. The sporangia measure 1.6−2.0 mm high and 1.0−1.4 mm wide. The dehiscence line along the distal sporangial margin divides sporangia into two possibly equal valves. The sporangial stalk seems to be very short, *ca* 0.7 mm long (see electronic supplementary material, figure S3*c*, lower arrow). A thin rim runs along the margin of the valves (see electronic supplementary material, figure S3c, upper arrow; S3*d*, arrow).

### (c) Affinity

The architecture of our plant, with leafless axes and lateral sporangia forming a terminal spike, recalls the members of the Zosterophyllopsida. This group has been long recognized as a unique subdivision of early vascular plants [37]; however, its monophyly has been frequently questioned by cladistic analyses [11,30]. The taxa included in different analyses were different, and thus no consensus has been reached. Nevertheless, some studies tentatively suggested a monophyletic clade of zosterophyllopsids, mostly following traditional taxonomic treatments [13].

Up to now, at least 37 genera assignable to the Zosterophyllopsida have been reported from localities worldwide, spanning the Silurian to Late Devonian. Most genera are distinguishable from our plant by their distinctive characters, such as enations, circinate tips and rowed arrangement of sporangia (see electronic supplementary material, table S1). *Macivera* from the Ludlow deposits of Bathurst Island, Canada, is characterized by dichotomized axes and tiny terminal spikes with clusters of sporangia [29]. Our plant is similar to *Macivera* in the small size of ts fertile regions with 2−10 sporangia, and the shape and thin rim of sporangia. However, in *Macivera,* no K-shaped branching was found and the terminal axes supporting fertile regions are quite short, differing from our specimens and many other zosterophyllopsids.

Our plant is clearly assignable to the genus *Zosterophyllum*. The generic diagnosis of *Zosterophyllum* was originally given as being ‘aquatic plants with creeping stems, from which arise narrow dichotomous branches. An ovoid or spherical sporangium produced on short pedicels, without subtending bracts, from a single axis, the whole forming a loose spike’ [17]. However, the original diagnosis is problematic owing to the lack of a clear apomorphic defining feature [38,39]. Later, Kotyk *et al*. [29] and Edwards [39] emphasized defining characters for this genus. Our material shows smooth axes with K-shaped branching, compact spike and sporangia inserted on short stalks, which are evidently assignable to *Zosterophyllum*. The genus *Zosterophyllum* is further divided into two subgenera *Zosterophyllum* and *Platyzosterophyllum* based on sporangial arrangement [40,41]. Because of the spiral arrangement of the sporangia, our plant belongs to the subgenus *Zosterophyllum*.

In contrast with most species of the subgenus *Zosterophyllum*, such as *Zosterophyllum deciduum*, *Zosterophyllum dushanense*, *Z. myretonianum*, *Zosterophyllum ovatum*, *Zosterophyllum ramosum*, *Z. shengfengense*, *Z. sinense* and *Zosterophyllum tenerum*, our plant exhibits a remarkably compact spike that distinguishes it from the above species (see electronic supplementary material, table S2). Meanwhile, a distinctly thickened border to the sporangium is absent in our plant but present in *Z. australianum*, *Zosterophyllum bifurcatum*, *Z. confertum*, *Zosterophyllum minifertillum*, *Z. rhenanum*, *Zosterophyllum yunnanicum* and *Z. qujingense*.

Our plant is similar to *Z. xishanense* and *Z. minorstachyum*, which were respectively collected from the Lower Devonian Xiaxishancun and Xitun formations of Qujing, Yunnan Province. However, the sizes of the axes, spikes and sporangia of the present specimens are much smaller than those of *Z. xishanense* and *Z. minorstachyum* (see electronic supplementary material, table S3). Our material and those of another species, *Zosterophyllum minor*, bear the smallest sized spikes in *Zosterophyllum*. In addition, sporangia in our material are oval and semicircular in shape, different from those of *Z. xishanense* (round in shape) and *Z. minorstachyum* (elliptical or round in shape). Considering the above comparisons, we assign our material to a new species, *Z. baoyangense* sp. nov.

### (d) Morphometric variations of *Zosterophyllum* species

**Figure 2.**
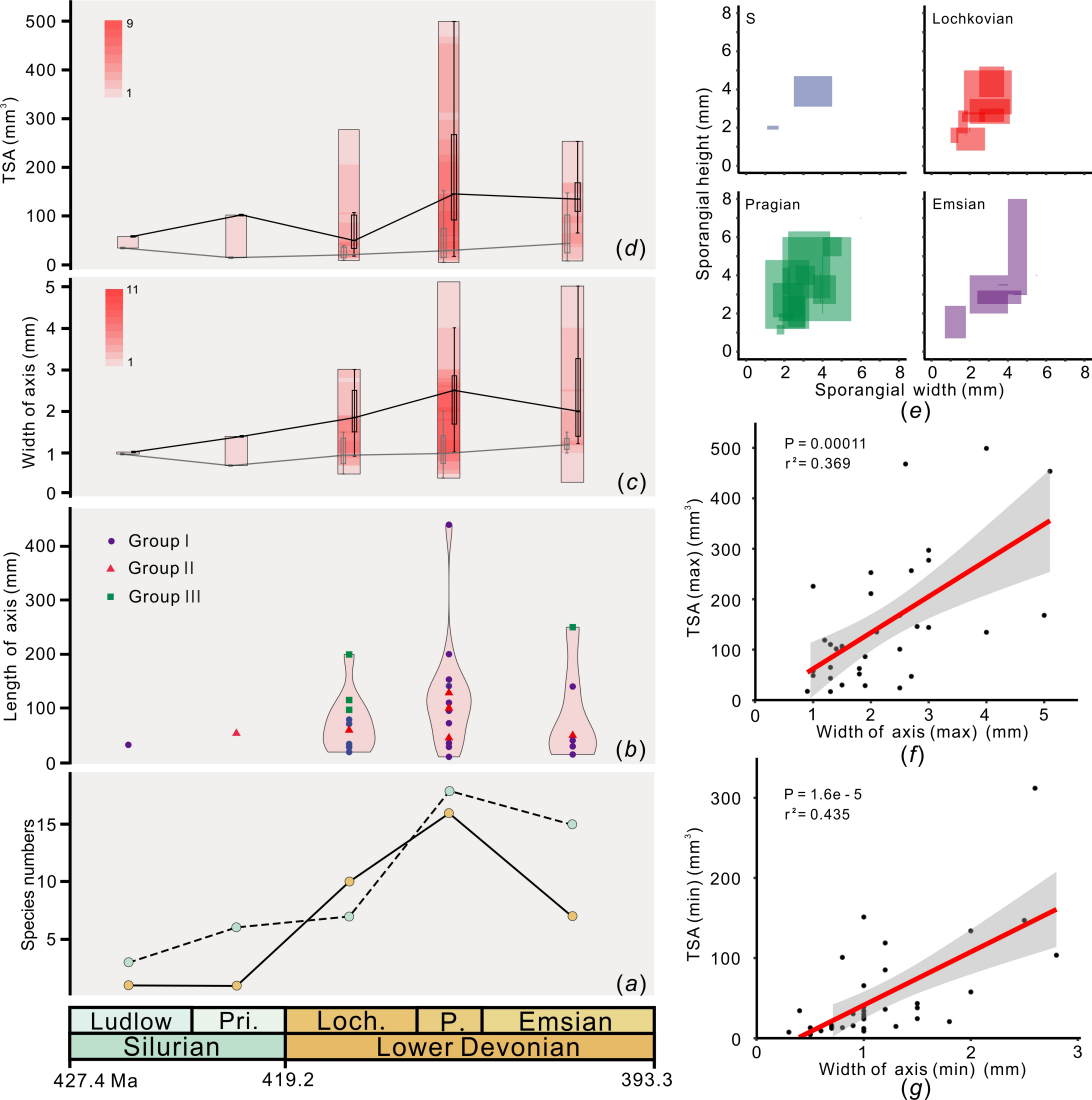
Diversity and morphology of *Zosterophyllum* species through the late Silurian to Early Devonian. (*a*) Species richness; solid and dashed lines based on data from our dataset and those of Cascales-Miñana and Meyer-Berthaud [20], respectively; (*b*) maximum length of axes; Groups I, II and III represent specimens with different preservation status (see §2 for details); (*c*) width of axes; the rectangles represent range of axial width of the sample taxa for each time bin; within each rectangle, the range of axial width for each taxon is plotted, and the density of such data is shown by the variation in colour intensity; the left and right inset boxplots in each rectangle represent, respectively, statistics of the minimum and maximum values of axial width for the sampled taxa; for boxplots, lines in the box are median values, boxes are 25%−75% quartiles and upper and lower values are range; same in figure 2*d*; (*d*) TSA; (*e*) Crossplot of sporangial width and height of different time bins; (*f*) Crossplot of maximum value of TSA and maximum axial width for all sampled *Zosterophyllum* species; the grey area indicates the 95% confidence interval; (*g*) Crossplot of minimum value of TSA and minimal axial width for all sampled *Zosterophyllum* species; abbreviations: TSA,total sporangial accommodation; S, Silurian; Pri., Pridoli; Loch., Lochkovian; P., Pragian; max, maximum; min, minimum.

The earliest stratigraphic record of *Zosterophyllum* was found in Silurian (Ludlow) deposits, and then species numbers gradually increased, finally reaching maximum diversity in the Lower Devonian [20]. Species diversity attained its maximum during the Pragian (figure 2*a*), and thenceforward numbers declined during the Emsian (figure 2*a*).

**Figure 3.**
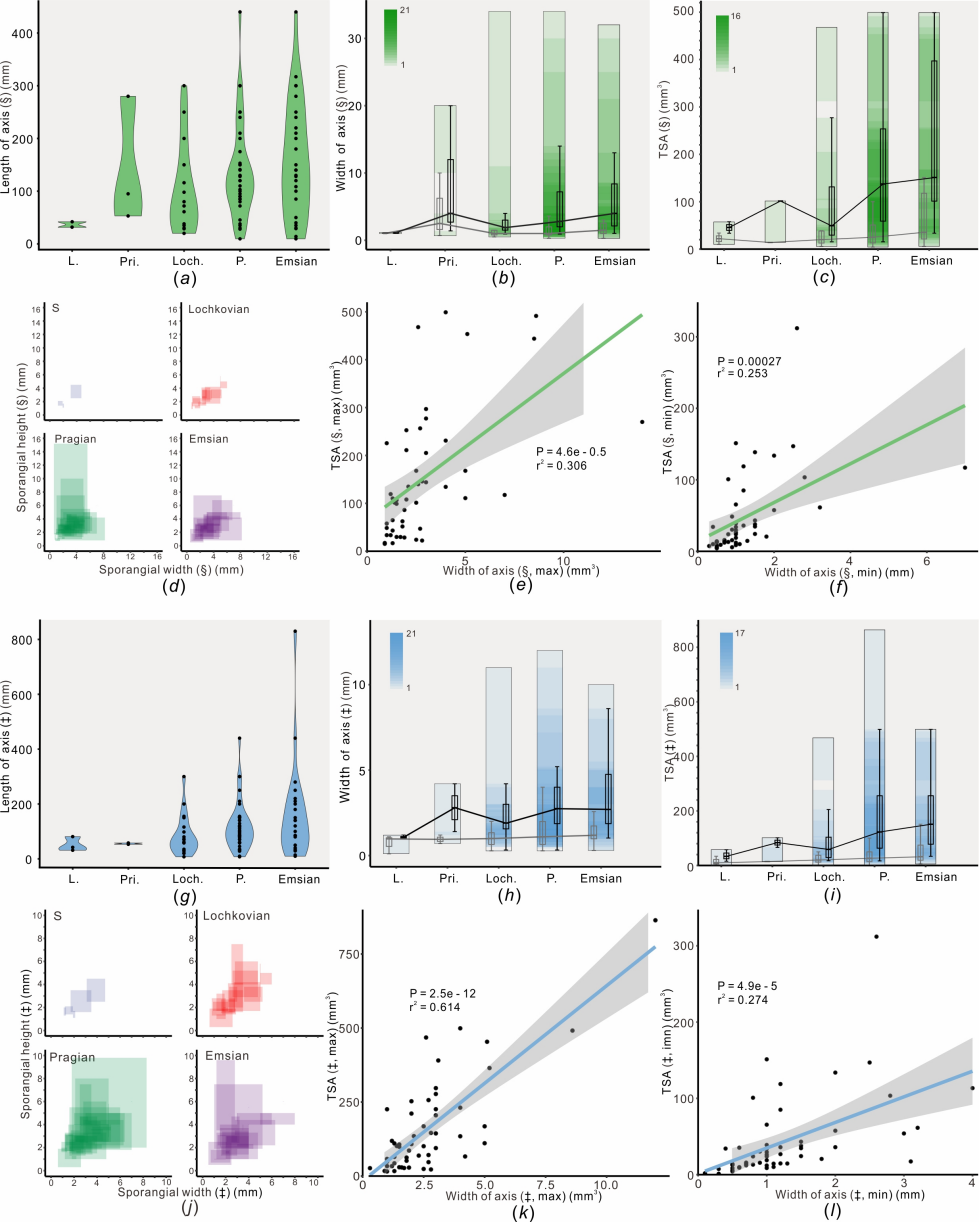
Morphological evolution of members of the Lycophytina *sensu* Kenrick & Crane and the Zosterophyllopsida *sensu* Hao & Xue through the late Silurian to Early Devonian. (*a–f*) Lycophytina *sensu* Kenrick & Crane, indicated by §; (*g–l*) Zosterophyllopsida *sensu* Hao & Xue, indicated by ‡; for each group, the figures show the maximum preserved length of axes (*a,g*), width of axes (*b,h*), TSA (*c,i*), crossplot of sporangial width and height of different time bins (*d,j*), crossplot of maximum value of TSA and maximum axial width for all sample taxa (*e,k*), and crossplot of minimum value of TSA and minimal axial width for all sample taxa (*f,l*). For boxplots in (*b*,*c*,*h*,*i*), lines in the boxes are median values, boxes are 25%−75% quartiles and upper and lower values are range. Abbreviations same as in figure 2.

Several morphological parameters were considered (see electronic supplementary material, figure S2), including width and length of axes, and size of spikes, etc. Considering that the axial length can be easily influenced by preservation condition, we divide the axes described in the literature into three groups (Groups I, II and III) to reduce the influence of taphonomy. The results show that differently preserved axes have a similar evolutionary pattern (figure 2*b*).

Two species reported from the Silurian, *Zosterophyllum* sp. and *Z. qujingense*, show preserved axial lengths of *ca* 31.4 and 53.0 mm, respectively, and the maximum axial widths are 1.0 and 1.4 mm, respectively (figure 2*b*,*c*). The preserved axial length of *Zosterophyllum* increases in the Lochkovian, ranging from 20.0 to 200.0 mm (average value = 74.1 mm, median value = 66.5 and *n* = 10). The well-preserved specimens of *Z. xishanense* and *Z. shengfengense*, both of Lochkovian age, exhibit a complete or near complete height, reaching up to 116.0 and 98.0 mm, respectively [16,42]. The Lochkovian axes of *Zosterophyllum* are generally wider than the Silurian ones (figure 2*c*). Among the known Lochkovian species, the maximum width of the axes reaches up to 3.0 mm in *Zosterophyllum fertile* and *Z. myretonianum*. The preserved axial length in the Pragian varies from 9.6 to 440.0 mm (average value = 120.1 mm, median value = 103.0 mm and *n* = 15). *Z. baoyangense* sp. nov. is represented by a nearly or almost complete specimen, only 45.4 mm long and 0.5−1.3 mm wide, which is the smallest taxon in the genus. During the Pragian, *Z. confertum* reaches 5.1 mm in width and up to 440 mm in preserved length. Compared with the decline in diversity of *Zosterophyllum* in the Emsian, the dimensions of plant axes show a wide range of values, from 14.0 to 250.0 mm long (average value = 87.2 mm, median value = 45.0 mm and *n* = 6) and 0.3 to 5.0 mm wide (figure 2*b*,*c*). The median value of minimum axial width shows a subtle increase from the Pridoli to Early Devonian, while the median value of maximum axial width reaches a peak in the Pragian (figure 2*c*).

The evolutionary trend of TSA for *Zosterophyllum* is similar to the change in axis size from the late Silurian to Early Devonian (figure 2*d*). For the Silurian species, the TSA of *Z. qujingense* ranges from 14.0 to 101.5 mm^3^, and the TAS of *Zosterophyllum* sp. from Bathurst Island of Canada ranges from 33.9 to 57.8 mm^3^. Along with the increase in diversity and size of species in the Lochkovian, the TSA increases remarkably, ranging from 8.7 to 277.2 mm^3^. The TSA attains its maximum variation during the Pragian, varying from 4.3 to 499.0 mm^3^. The minimum value of the TSA is represented by *Z. baoyangense* sp. nov., at only 4.3 to 16.8 mm^3^, while the TSA of *Z. australianum* is 29.6 to 499.0 mm^3^—which is at least six times larger than the former. During the Emsian, the TSA began to decline, ranging from 7.4 to 252.7 mm^3^ (figure 2*d*). The median value of minimum TSA for *Zosterophyllum* species shows subtle changes from the Pridoli to Early Devonian, while the median value of maximum TSA shows a peak in the Pragian (figure 2*d*). From Silurian to Pragian, the range of sporangial size gradually increases, finally reaching the largest occupation of morphological space in the Pragian, in the two-dimensional space of sporangial height and width (figure 2*e*). The TSA seems to be closely related to axial width (figure 2*f*,*g*), when considering all *Zosterophyllum* species, that is, larger species have larger TSA values.

### (e) Evolution of sporangium size and TSA in the L-KC and the Z-HX

For the members of L-KC and Z-HX, the length and width of axes generally follow the same trend of evolution, increasing from the Ludlow to Early Devonian, with the maximum range being reached in the Pragian or Emsian (figure 3*a*,*b*,*g*,*h*). The TSA values of the members of L-KC and Z-HX also show a similar pattern, steadily increasing from the Ludlow to the Early Devonian, and the maximum range of TSA is attained in the Pragian (figure 3*c*,*i*). Comparable to *Z. baoyangense* sp. nov., whose TSA is *ca* 4.3–16.8 mm^3^, two other plants show the lower extreme values of TSA: the TSA of *Macivera gracilis*, one of the earliest members of the Zosterophyllopsida, is only *ca* 1.7–15.6 mm^3^; and the TSA of *Gippslandites minutus*, a zosterophyllopsid reported from the Lochkovian–Pragian of Victoria, Australia, *ca* 1.0–26.7 mm^3^. For the members of L-KC, the range of their sporangium size was limited in the Silurian, began to radiate during the Lochkovian, attained the maximal values in the Pragian and then declined in the Emsian (figure 3*d*); and a similar pattern is observed in the members of Z-HX (figure 3*j*). The TSA values are positively correlated with the width of axes in different plant groups, i.e. either in the L-KC or in the Z-HX (figure 3*e*,*f*,*k*,*l*).

## 4. Discussion

Both vegetative, clonal growth and sexual reproduction occurred in early land plants [3–5]. It has been suggested that the former was employed to overcome the problems caused by weakly developed vascular tissues [3] or to survive in environments with frequent disturbance, such as sediment burial [5,6]. Early Devonian *Nothia* from the Rhynie Chert shows how both clonal growth and sexual reproduction strategies worked in an individual plant to enable it to survive and expand its habitats; and its repeated ramet consisted of a rhizome bearing a ventral rhizoidal ridge and a branched, aerial fertile shoot [4]. Similar clonal strategies have also been demonstrated in other Rhynie plants, such as *Aglaophyton* and *Rhynia* [4,43]. Nevertheless, sexual reproduction was also very common in *Zosterophyllum* and the zosterophyllopsids, as is evidenced by the presence of ubiquitous sporangia. Here we focus on their divergent strategies of sexual reproduction.

*Zosterophyllum* lacks leaves or leafy structures and has no spines or appendages on its axes. Well-preserved species such as *Z. shengfengense*, *Z. myretonianum* and *Z. sinense* demonstrate that the sporophytes of this group are photosynthetically autonomous without thallus-like organs [16,21,22]. Thus, the axes are the main photosynthetic organs of the plant, with stomata on the surface [44,45]. Therefore, it is reasonable to assume that the photosynthetic output of *Zosterophyllum* depends on its axial width and length. Larger and more robust axes will increase the photosynthetic area and finally gain more energy. For example, each 1 mm increase in axial diameter (Δ*R*) will give rise to 31.4 mm^2^ (Δ*S*) increase in photosynthetic area (surface area of axis) for the same 10 mm-long axis (*h*) (Δ*S* = *π*·Δ*R·h*). In addition, the increase in axial length also adds to the photosynthetic area. Emergences occur in some zosterophyllopsids, probably contributing to photosynthesis [46], but *Zosterophyllum* species lack any spines. In fact, in addition to photosynthesis, the axes of plants had support and transport functions [47], and the realization of these functions would certainly cost a large amount of energy and nutrition to build necessary tissues, such as xylem, cortex and epidermis. During the Pragian, *Z. confertum* and *Z. baoyangense* sp. nov. exhibited huge differences in their size (the former reaches 5.1 mm in width and up to 440 mm in preserved length, while the latter is 0.5−1.3 mm wide and only 45.4 mm in preserved length), indicating their divergent investment strategies in vegetative growth. It is suggested that plant lifespan is usually positively related to plant size, from the tiniest phototrophs to the largest trees [48]. As a result, compared with *Z. confertum*, *Z. baoyangense* sp. nov. might have had a shorter lifespan, and it tended to invest fewer resources in vegetative growth.

**Figure 4.**
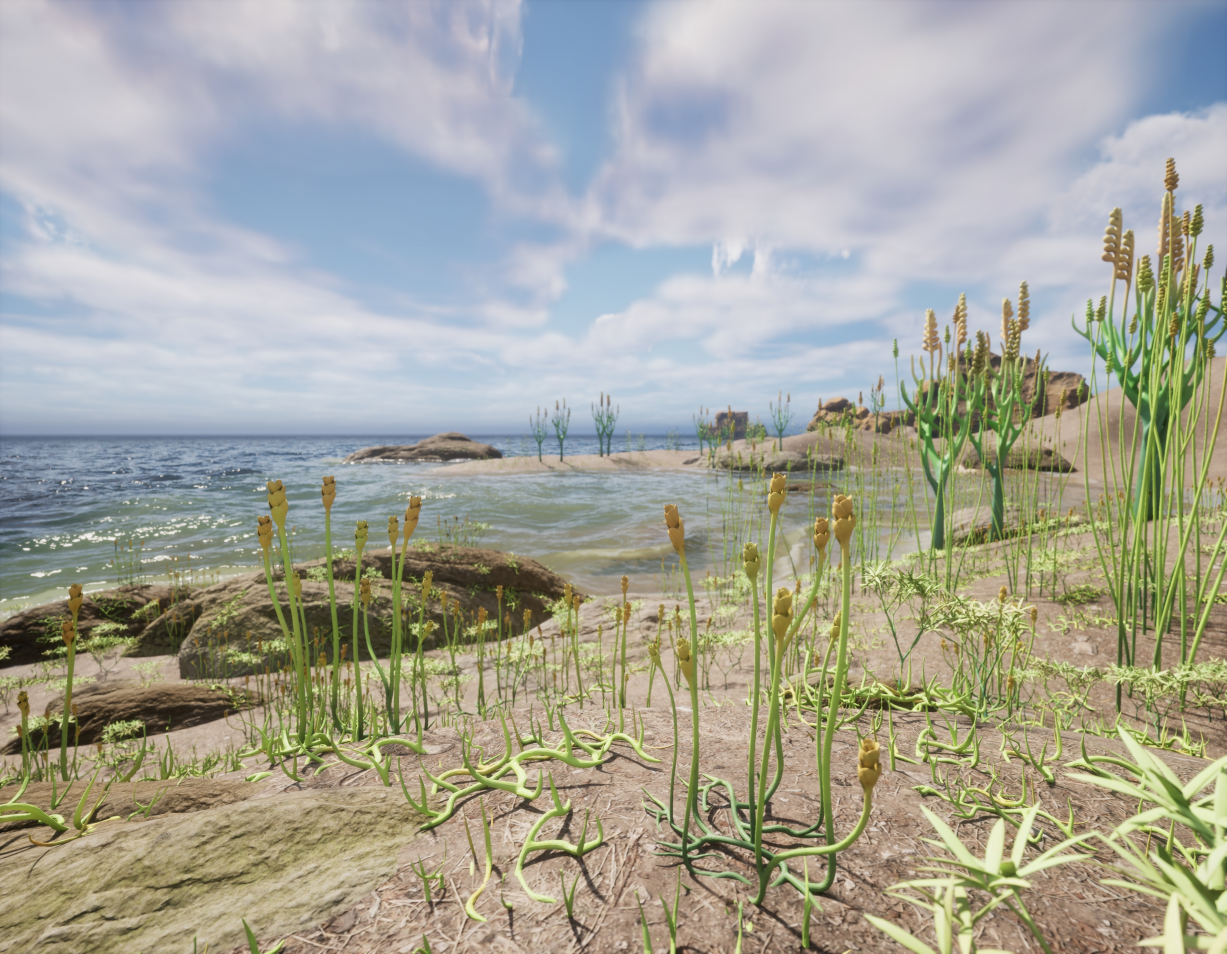
Artist’s restoration of part of the Early Devonian Mangshan flora, with plant communities of *Zosterophyllum baoyangense* sp. nov. at the front, and *Teyoua antrorsa*, *Zosterophyllum australianum* and an unnamed zosterophyllopsid to the back.

The TSA is regarded as an index of reproductive output/investment to assess the total cost of reproductive organs, although it is affected by many other factors, such as taphonomy and sampling intensity. The larger value of TSA in *Z. australianum* (133.8−211.1 mm^3^) is quite distinct from the smaller value of *Z. baoyangense* sp. nov. (4.3−16.8 mm^3^), and interestingly these two species occur in the same beds of the Baoyang section (see electronic supplementary material, figure S1; figure 4). The TSA of *Z. australianum* is 12–31 times larger than that of *Z. baoyangense* sp. nov. Thus, huge differences of TSA existed in the Pragian that are surely not caused by taphonomy factors.

On a broader scale, differences of TSA also exist in the zosterophyllopsids. The maximum TSA reaches 864.0 mm^3^ in the Pragian within the members of Z-HX, represented by *Bathurstia denticulata*, whose axes are 4.0−12.0 mm wide, while for *Gippslandites minutus* and *Z. baoyangense* sp. nov.—which were also in the Pragian and whose axes are only 0.3−1.3 mm wide—the minimum TSA values are only 1.0 and 4.3 mm^3^, respectively (figure 3*h*,*i*; see electronic supplementary material, table S4). Such great differences in TSA indicate the differences in total output of reproductive organs. More robust plants have more photosynthetic products to support the development of more reproductive output, whereas tiny plants usually have less reproductive output (figures 2*f*,*g*; 3*e*,*f*,*k*,*l*). In conclusion, the smaller zosterophyllopsid plants usually have a shorter lifespan with lower reproductive output, while the larger zosterophyllopsids have a longer lifespan with higher reproductive output.

Plants usually contend with limited resources in their growth environments, such as shortages of light, water and critical limiting elements including nitrogen (N) and phosphorus (P) [49], and one solution is to adopt divergent life histories [50]. The various life-history strategies adopted by modern plants were divided into two groups by MacArthur & Wilson [51], that is, *r*-selected and *K*-selected species. The *r*-selected species are characterized by rapid development, high maximal intrinsic rate of natural increase, early reproduction, small body size, semelparity (reproduction once in a lifetime) and short lifespan, and they are represented by ephemerals in desert plants [52]. On the other hand, *K*-selected species tend to show slower development, greater competitive ability, lower resource thresholds, delayed reproduction, larger body size, iteroparity and longer lifespan [52], such as the conifer *Sequoiadendron giganteum* [53]. In addition, *r*-selected species have higher reproduction allocation than *K*-selected species. The *r*-selected and *K*-selected species are two extremes of the *r–K* continuum [52]. *Z. baoyangense* sp. nov. resembles the *r*-selected species, inferred by its small body size and probable short lifetime. The alternative strategy is seen in the comparatively large species such as *Z. confertum* and *B. denticulata*, which show enhanced reproductive ability by increasing sporangial numbers and size, although probably at the cost of delayed reproduction, compared with *Z. baoyangense* sp. nov.

Considering the intensities of factors such as stress and disturbance, three-strategy models were provided by Grime [50,54], including competitive (*C*), stress-tolerant (*S*) and ruderal plants (*R*); the ruderals are characterized by small stature, rapid life cycle, short longevity of leaf and other traits [50]. *Z. baoyangense* sp. nov. shows small stature, probable rapid lifespan and short longevity of photosynthetic organs and seems to be assignable to the category of ruderal plants. Admittedly, some physiological traits, such as phenology of leaf production and flowering in ruderal plants, are either inapplicable or cannot be observed in fossil plants. Both *r*-selected plants and ruderals usually complete their whole lifespans rapidly, and they tend to reproduce rapidly when they are experiencing high disturbance.

The evolutionary history of vascular plants can be divided into five Evolutionary Floras through geological time, that is, the Rhyniophytic Flora (or Eotrachophytic, dominated by rhyniophytes and cryptospore producers), Eophytic Flora (this concept follows Cleal & Cascales-Miñana [55]; dominated by zosterophyllopsids), Palaeophytic Flora, Mesophytic Flora and Cenophytic Flora [55]. The Early Devonian witnessed the transition from the Rhyniophytic to the Eophytic Evolutionary Floras [55]. Morris & Edwards [56] described the vegetational change in the Lower Devonian of the Welsh Borderland and showed that the previously dominant rhyniophytes were replaced by more competitive zosterophyllopsids. Some sedimentological and fossil evidence supported the idea that rhyniophytes and cryptospore producers survived in dry, dynamic landscapes owing to their smaller size resulting in short life cycles [57], but another view is that they were better adapted to wet environments subject to disruption by flooding [58]. It has been suggested that most zosterophyllopsids, which are usually larger than rhyniophytes, lived in more stable environments to achieve their full growth potential [57,58]. However, dryland river systems with seasonal wet–dry cycles were recognized in the Lower Devonian of South China [59], where *Z. myretonianum* and cf. *Zosterophyllum* sp. were found [60,61]. *Z. baoyangense* sp. nov. is an extremely small plant among the known zosterophyllopsids, close in size to some Lower Devonian rhyniophytes such as *Jiangyounia gengi* [32]. We argue that the diversification of zosterophyllopsids, particularly of the genus *Zosterophyllum*, as a major component of the explosion of vascular plants in the Early Devonian [13,20] might have been driven by the evolution of varied life-history strategies.

## 5. Conclusion

The zosterophyllopsids, and especially their type genus *Zosterophyllum*, are prominent components of Early Devonian floras. Herein we report a new species, *Z. baoyangense* sp. nov., from the Lower Devonian (Pragian) of Guizhou Province, southwestern China, which exhibits the smallest sized plant body in this genus and bears tiny spikes. We investigated TSA, which is here defined as a new descriptor, of *Zosterophyllum* species and on a broader scale, of the Lycophytina *sensu* Kenrick & Crane and the Zosterophyllopsida *sensu* Hao & Xue. The results reveal a gradual increase in the range of TSA values in these groups from late Silurian (Ludlow) to Early Devonian (Emsian). The smaller values of TSA in *Z. baoyangense* sp. nov. and two other zosterophyllopsids (*M. gracilis* and *G. minutus*) indicate that these plants may be similar to *r*-selected species in their life-history strategy. Higher values of TSA are present in other *Zosterophyllum* species and other genera. We argue that the transition from Rhyniophytic Flora to Eophytic Flora was probably driven by the evolution of divergent life-history strategies in *Zosterophyllum* and more broadly in zosterophyllopisds generally.

## Data Availability

The supplementary figures and tables, and the R code to process the data and to reproduce all the figures, are available online in the Dryad Digital Repository [36]. Supplementary material is available online [62].
